# Evaluation of Antifungal Activity of *Naja pallida* and *Naja mossambica* Venoms against Three *Candida* Species

**DOI:** 10.3390/toxins12080500

**Published:** 2020-08-04

**Authors:** Ewelina Kuna, Aleksandra Bocian, Konrad K. Hus, Vladimir Petrilla, Monika Petrillova, Jaroslav Legath, Anna Lewinska, Maciej Wnuk

**Affiliations:** 1 Department of Biotechnology, Institute of Biology and Biotechnology, University of Rzeszow, 35-310 Rzeszow, Poland; gawel.ewelina@gmail.com; 2 Department of Biotechnology and Bioinformatics, Faculty of Chemistry, Rzeszow University of Technology, 35-959 Rzeszow, Poland; bocian@prz.edu.pl (A.B.); knr.hus@gmail.com (K.K.H.); jaroslav.legath@uvlf.sk (J.L.); 3 Department of Physiology, University of Veterinary Medicine and Pharmacy, 041 81 Kosice, Slovak Republic; vladimir.petrilla@uvlf.sk; 4 Zoological Department, Zoological Garden Kosice, 040 06 Kosice, Slovak Republic; 5 Department of General Education Subjects, University of Veterinary Medicine and Pharmacy, 041 81 Kosice, Slovak Republic; monika.petrillova@uvlf.sk; 6 Department of Pharmacology and Toxicology, University of Veterinary Medicine and Pharmacy, 041 81 Kosice, Slovak Republic

**Keywords:** *Candida albicans*, cobra venoms, phospholipase secretion, ammonia signaling, colony development and aging

## Abstract

In contrast to comprehensively investigated antibacterial activity of snake venoms, namely crude venoms and their selected components, little is known about antifungal properties of elapid snake venoms. In the present study, the proteome of two venoms of red spitting cobra *Naja pallida* (NPV) and Mozambique spitting cobra *Naja mossambica* (NMV) was characterized using LC-MS/MS approach, and the antifungal activity of crude venoms against three *Candida* species was established. A complex response to venom treatment was revealed. NPV and NMV, when used at relatively high concentrations, decreased cell viability of *C. albicans* and *C. tropicalis*, affected cell cycle of *C. albicans*, inhibited *C. tropicalis*-based biofilm formation and promoted oxidative stress in *C. albicans*, *C. glabrata* and *C. tropicalis* cells. NPV and NMV also modulated ammonia pulses during colony development and aging in three *Candida* species. All these observations provide evidence that NPV and NMV may diminish selected pathogenic features of *Candida* species. However, NPV and NMV also promoted the secretion of extracellular phospholipases that may facilitate *Candida* pathogenicity and limit their usefulness as anti-candidal agents. In conclusion, antifungal activity of snake venoms should be studied with great caution and a plethora of pathogenic biomarkers should be considered in the future experiments.

## 1. Introduction

The incidence of fungal infections in humans is rapidly increasing worldwide [1]. The widespread use of modern medical treatments such as broad-spectrum antibiotics, anti-cancer and immunosuppressive therapies and invasive surgical procedures may be implicated in increased number of individuals who are vulnerable to fungal infections [1,2]. *Candida albicans*, a polymorphic fungus, is considered to be the most prevalent human fungal commensal [1,2]. *C. albicans* is able to asymptomatically colonize the gastrointestinal tract, female reproductive tract, oral cavity and skin of 30–70% of healthy individuals [3]. However, *C. albicans* is also a pathogen causing superficial mucosal infections such as vulvovaginal candidiasis and oropharyngeal candidiasis, dermal infections and life-threatening disseminated candidiasis and fungemia [1,2,3]. The evolutionary success of *C. albicans* is based on its morphological, biochemical and genetic flexibility that promotes adaptation to a wide range of host niches [3,4]. *C. albicans* developed a number of survival strategies that may facilitate its pathogenic potential in the human body [1,3,4,5]. Pathogenic features of *C. albicans* may rely on yeast-to-hyphae transition, white-to-opaque switching and mating, hyphae-associated expression of adhesins, invasion into host cells by induced endocytosis or active penetration, biofilm formation, release of hydrolases that stimulates the breakdown of tissues, acquisition of micronutrients from host cells using zinc and iron uptake systems and activation of stress response pathways conferring resistance to harsh environmental conditions, e.g., reactive oxygen species (ROS), reactive nitrogen species (RNS), low pH and nutrient limitation [1,3,4,5]. *C. albicans* is postulated to be the main cause of candidiasis; however, some of non-*C. albicans Candida* (NCAC) species such as *Candida glabrata*, *Candida parapsilosis*, *Candida tropicalis* and *Candida krusei* may also be implicated in *Candida* infections [1,3]. For example, *C. glabrata* may be more frequently isolated from infections in humans compared to *C. albicans* because of its intrinsic resistance to conventional fluconazole-based antifungal therapy [1,3]. *C. glabrata* and *C. tropicalis* are more frequently detected in patients with cancer and neutropenia; *C. krusei* infections are commonly observed in post- hematopoietic stem cell transplantation patients; *C. parapsilosis* infections are more common in neonates rather than adults; and *C. parapsilosis* is more frequently identified during catheter-related infections [3]. Several risk factors for the development of candidemia can be considered, namely extended hospitalization, central venous catheter, polytrauma, age, immunosuppression, neutropenia and cancer [3]. Treatment of systemic candidiasis might be challenging because of limited number of antimycotic agents available, antifungal drug resistance, lack of antifungal vaccines and some diagnostic difficulties [1,3,6]. Thus, more studies are needed for identification of new antifungal therapeutics that would attenuate the pathogenic potential and virulence traits of *Candida* species. For example, snake venoms can be considered as “mini-drug libraries” in which each drug may have potential pharmacological and therapeutical activity [7,8,9].

Snake venoms, highly complex cocktails of bioactive compounds, are composed of both protein (non-enzymatic proteins, peptides and enzymes with specific biological activities, namely proteolytic enzymes, arginine ester hydrolases, thrombin-like enzymes, hyaluronidases, phospholipase A_2_ (PLA_2_) fraction, acetylcholinesterases, nucleases and L-amino-acid oxidases (LAAO)) and non-protein compounds (carbohydrates, lipids, metal ions and unidentified substances) [7,8,9]. Snake venoms may exert a number of biological effects such as neurotoxicity, myotoxicity, cardiotoxicity, hemorrhage, pro- and anti-coagulation to facilitate feeding or defense by the producing animal [7]. Moreover, selected components of snake venoms may be also considered as new pharmaceuticals with high therapeutical potential such as FDA approved drugs based on snake venoms Captopril^®^ (Enalapril, an inhibitor of angiotensin-converting enzyme), Integrilin^®^ (Eptifibatide, glycoprotein IIb/IIIa inhibitor) and Aggrastat^®^ (Tirofiban, glycoprotein IIb/IIIa inhibitor) to treat hypertension, acute coronary syndrome and heart attack, respectively [8,9]. In contrast to well established antibacterial activity of crude snake venoms and selected components such as PLA_2_ and LAAO fractions [10,11,12], little is known about antifungal properties of snake venoms, especially elapid snake venoms (Elapidae family) [9]. Thus, it seems reasonable to study antifungal activity of cobra venoms more comprehensively.

In the present study, the proteome of two venoms from elapid snakes, namely red spitting cobra *Naja pallida* (NPV) and Mozambique spitting cobra *Naja mossambica* (NMV), was analyzed and the antifungal properties of crude venoms were comprehensively investigated using three *Candida* species: *C. albicans*, *C. glabrata* and *C. tropicalis.* The potential applications of NPV and NMV as anti-candidal agents are evaluated and discussed.

## 2. Results and Discussion

### 2.1. Proteomic Analysis of Naja pallida and Naja mossambica Venoms

**Figure 1:**
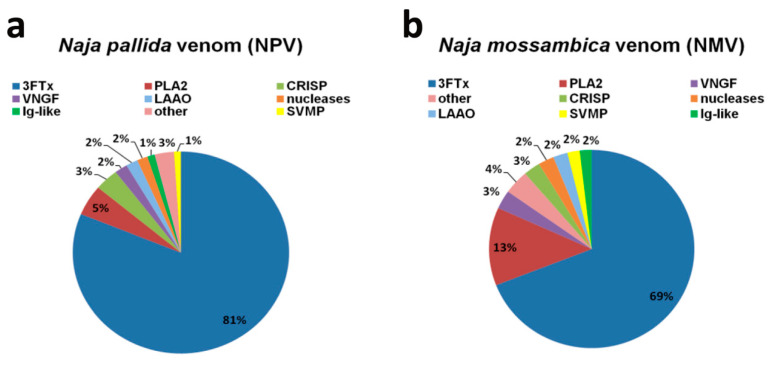
The quantitative analysis of two cobra venom proteins: *N. pallida* venom (NPV) (**a**); and *N. mossambica* venom (NMV) (**b**). The identified protein groups with protein content less than 1% of total protein were classified as “other”. Detailed description of “other” category can be found in the Supplementary Materials. Values have been rounded up to the nearest integer. 3FTx, three-finger toxin family; PLA_2_, phospholipase A_2_; VNGF, venom nerve growth factor; CRISP, cysteine-rich secretory protein; LAAO, L-amino acid oxidase; SVMP, snake venom metalloproteinase; Ig-like, Ig-like superfamily SSF48726.

LC-MS/MS analysis revealed that, in both analyzed cobra venoms, namely *N. pallida* venom (NPV) and *N. mossambica* venom (NMV), the main protein fraction is a three-finger toxin family (3FTx) (Figure 1).

3FTx accounts for about 81% and 69% of the total protein content of NPV and NMV, respectively (Figure 1). The second most abundant group is PLA_2_ fraction—5% in NPV and 13% in NMV (Figure 1). The remaining proteins, with the content higher than 1% of the total protein fraction, are VNGF (venom nerve growth factor), CRISP (cysteine-rich secretory protein), nucleases, LAAO, metalloproteinases (SVMP) and Ig-like proteins, and their levels are comparable in both cobra species (Figure 1). When considered as a combined fraction, these six groups represent 11% and 14% of total protein fraction in NPV and NMV, respectively (Figure 1). Moreover, small amounts of phosphodiesterases, Vespryn/ohanin proteins, serine proteases (SVSP), cysteine proteases (SVCP), cobra venom factor (CVF), phospholipase B and vascular endothelial growth factor (VEGF) can also be detected in both venoms (Supplementary Materials). However, only in NMV, Kunitz type protease inhibitor, phosphatase and type C lectin were observed, whereas, only in NPV, PLA_2_ inhibitor, hyaluronidase and von Willebrand factor (VWF) were noticed (Supplementary Materials). Detailed information on identification and quantitative analysis of NPV and NMV proteins can be found in Supplementary Materials.

The venom composition of both analyzed species is very similar and typical for the *Naja* genus with the abundance of 3FTx proteins [13,14,15,16]. In contrast, we observed a relatively lower content of PLA_2_ (Figure 1) compared to other cobra species that may be due to a different methodological approach used for protein quantification [17] and age-, sex- or season-related effects on venom composition [7,18]. Moreover, in contrast to previous report [14], we were able to detect protein groups such as CRISP, SVMP and LAAO (Figure 1). Surprisingly, Ig-like superfamily SSF48726 proteins with unknown function(s) were identified in both NPV and NMV (Figure 1). More recently, this protein category has been also reported to be present in *Naja ashei* venom [19]. A widespread Ig-like domain can be found in proteins that differ in cellular localization, amino acid composition and biological functions, namely in immunoglobulins, enzymes and their inhibitors, transcription factors and ion channels [19]. The function(s) of this protein family have to be determined, but the authors concluded that these proteins are not related to the immune system [19].

### 2.2. NPV- and NMV-Mediated Changes in Cell Viability, Cell Cycle and Biofilm Formation in Three Candida Species

The antibacterial activity of snake venoms both crude venoms and their components is well established [10,11,12]. However, antifungal properties of snake venoms are much less studied and the research is focused mainly on the biological activities of venoms of viperid species (Viperidae family) [20,21,22,23]. In contrast, the antifungal activity of the venoms of elapid species (Elapidae family) is less evident [24,25]. For example, it has been reported that the venoms of two cobras, namely *Walterinnesia aegyptia* and *Naja haje arabica*, have no antimycotic activity against the human fungal pathogen *C. albicans* based on zone of growth inhibition assay [24]. Thus, in the present study, we evaluated the biological activities of NPV and NMV against three *Candida* species, namely *C. albicans*, *C. glabrata* and *C. tropicalis* more comprehensively.

**Figure 2:**
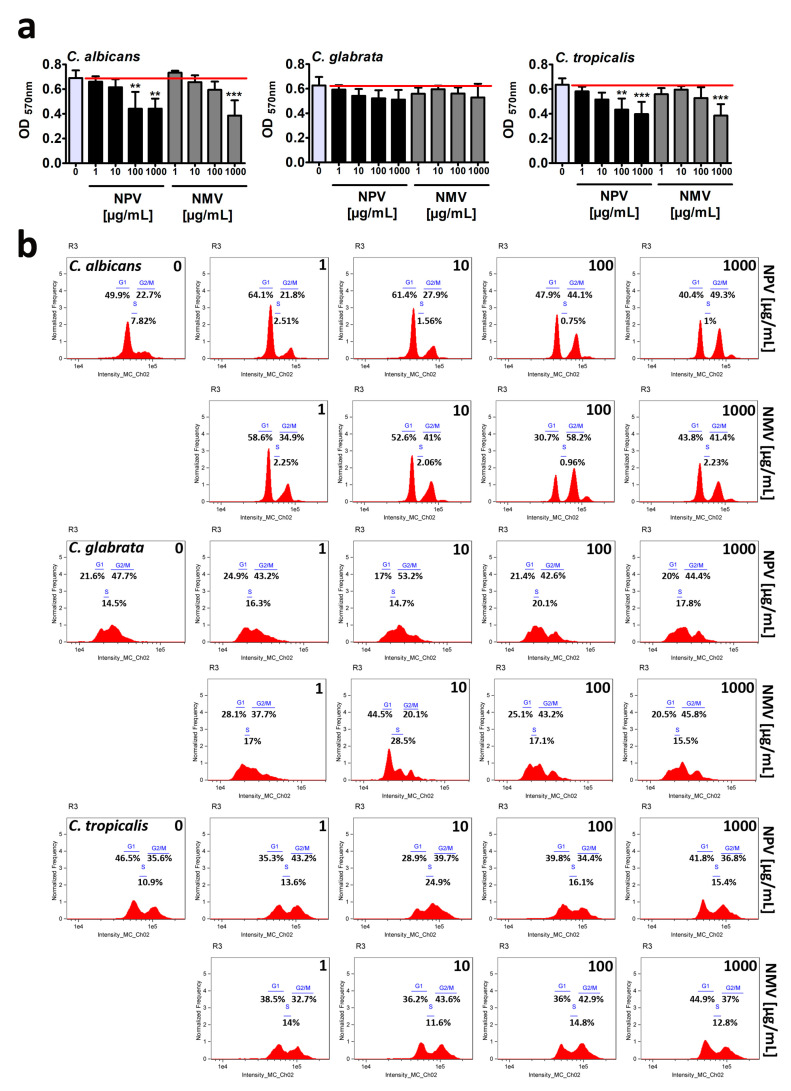
NPV- and NMV-mediated changes in cell viability (**a**) and cell cycle (**b**) of three *Candida* species. (**a**) Metabolic activity and cell viability were assessed using resazurin assay. To emphasize snake venom action, a red horizontal line is added. Bars indicate SD, *n* = 3, *** *p* < 0.001, ** *p* < 0.01 compared to the control (ANOVA and Dunnett’s a posteriori test). (**b**) DNA-based cell cycle analysis was conducted using an Amnis^®^ FlowSight^®^ imaging flow cytometer and IDEAS software. Representative histograms (normalized frequency vs intensity) are presented. NPV, *N. pallida* venom; NMV, *N. mossambica* venom.

Firstly, resazurin-based metabolic activity, an indicator of cell viability, was analyzed upon the treatments with crude NPV and NMV for 2 h (Figure 2a). Four concentrations of NPV and NMV were selected, namely 1, 10, 100 and 1000 µg/mL (Figure 2a). We were interested if both low (1 µg/mL) and high (1 mg/mL) concentrations of cobra venoms might have antifungal activity (here, cytotoxic effects). Moreover, we wanted to reveal if short-term treatment (here 2-h incubation) might stimulate cytotoxicity. The metabolic activity of *C. glabrata* cells were not affected even when high concentration (1 mg/mL) of NPV and NMV was used (Figure 2a). In contrast, 2-h stimulation with NPV and NMV decreased cell viability of *C. albicans* and *C. tropicalis* (Figure 2a). However, the inhibitory effects were observed when relatively high concentrations were considered, namely 100 and 1000 µg/mL (Figure 2a). The differences of cobra venom-induced cytotoxic effects in three *Candida* species may be due to diverse genetic backgrounds of fungal isolates. In both *Candida* species, namely *C. albicans* and *C. tropicalis,* NPV was found to be more active than NMV (Figure 2a). It is postulated that metalloproteinases, PLA_2_ and crotamine isolated from viperid snake venoms may possess antifungal properties [9]. However, the levels of PLA_2_ and SVMP in NPV were not higher than in NMV (Figure 1). Thus, one cannot conclude that increased sensitivity to NPV treatment is mediated by PLA_2_ and SVMP (Figure 1 and Figure 2a). The sensitivity to cobra venom treatment was comparable in two *Candida* species, e.g., stimulation with NMV at the concentration of 1000 µg/mL resulted in a decrease in metabolic activity of about 44% and 39% in *C. albicans* and *C. tropicalis*, respectively (*p* < 0.001, Figure 2a).

Secondly, NPV- and NMV-mediated cell cycle progression was evaluated (Figure 2b). The phases of cell cycle were the most affected in *C. albicans* cells subjected to crude venom treatment at the concentrations of 100 and 1000 µg/mL (Figure 2b). NPV and NMV promoted cell cycle arrest at the G2/M phase in *C. albicans* (Figure 2b). An increase of about 20–35% in *C. albicans* cell fraction that stayed at the G2/M phase was revealed upon stimulation with 100 and 1000 µg/mL cobra venoms (Figure 2b). This may suggest a specific antiproliferative activity of NPV and NMV against *C. albicans* cells (Figure 2b). Cobra venom-mediated effects on cell cycle of *C. glabrata* and *C. tropicalis* were mild to moderate (Figure 2b). Except for 10 µg/mL NMV-induced G1 cell cycle arrest of *C. glabrata*, the effects were relatively slight (Figure 2b). As mentioned above, data on crude cobra venom-related effects on fungal cell growth and cell cycle progression are limited [24]. More recently, cardiotoxin 1 (CTX-1), a component of the venom of the Chinese cobra *Naja atra*, has been shown to be active against several unicellular fungal species, namely *C. albicans*, *C. glabrata* and *Malassezia pachydermatis* (minimal fungicidal concentration (MFC) ranging from 6.3 to 50 μg/mL) [25]. Much more data are available when considering the antifungal activity of the venoms from the snake family Viperidae [20,21,22,23]. The crude venom of the Lebanon viper *Montivipera bornmuelleri* slightly reduced the growth of *C. albicans* and had no effect on other fungi, namely *Aspergillus flavus* and *Penicillium digitatum* [21]. In contrast, the venom of the Ottoman viper *Montivipera xanthina* exhibited potent activity against *C. albicans* ATCC 10239 (minimal inhibitory concentration (MIC) of 7.8 μg/mL and MFC of 62.5 μg/mL) [22]. The crude venoms from two Amazonian snakes, namely *Bothrops atrox* and *Crotalus durissus ruruima* (Viperidae family), had limited antifungal activity against two *C. albicans* strains [23]. The venoms did not affect the growth of *C. albicans* ATCC 36232, whereas slight inhibitory effects (8–9% growth inhibition) were observed against the growth of *C. albicans* KL-07 when the venom concentrations of 200 and 400 μg/mL were considered, respectively [23]. The antifungal activity of the venom of the South American rattlesnake *Crotalus durissus cumanensis* has been also evaluated against fourteen yeast and ten mold fungal isolates, such as several *Candida, Penicillium*, *Aspergillus* and *Fusarium* species, *Cryptococcus neoformans* and *Sporothrix schenckii* [20]. According to well diffusion data, when 400 µg of *C. durissus cumanensis* venom were applied into the test well, the most susceptible to venom treatment were *C. albicans* 90028, *C. parapsilosis* 312 and *S. schenckii* 21076 with 15, 15 and 14 mm of zone of growth inhibition, respectively [20].

It is widely accepted that venom peptides from the group of antimicrobial peptides (AMPs) have antibacterial properties [12]. However, it is suggested that AMPs may also exert some antifungal effects such as Pep5Bj peptide from *Bothrops jararaca* venom (Viperidae family) [26], crotamine from *Crotalus durissus terrificus* venom (Viperidae family) [27,28] and cathelicidin from the banded krait *Bungarus fasciatus* venom (Elapidae family) [29]. However, we were unable to identify AMPs in both NPV and NMV (in this study). In contrast, PLA_2_ fraction and LAAOs with well-documented antibacterial properties [12,30] were detected in both analyzed venoms, namely NPV and NMV (PLA_2_ is the second most abundant fraction and the content of LAAOs is 2% of total proteins, in this study). Perhaps, PLA_2_ and LAAOs may also account for some antifungal effects observed in this study. Indeed, BmarLAAO, LAAO isolated from *Bothrops marajoensis* (Viperidae family), inhibited the growth of *C. albicans* as judged by a radial diffusion assay [31]. LAAO from the Malayan pit viper *Calloselasma rhodostoma* (Viperidae family) promoted apoptotic cell death in the budding yeast *Saccharomyces cerevisiae* [32]. This cytotoxic effect was mediated by hydrogen peroxide produced by the enzymatic activity of LAAO [32]. Moreover, pEM-2, a 13-mer synthetic peptide variant derived from myotoxin II, a phospholipase A_2_ homologue present in the venom of *Bothrops asper* (Viperidae family), exerted potent antifungal effects in *C. albicans* SC5314 strain [33]. pEM-2, when added at the concentration of 1 µg/mL for 90 min, diminished the ability to form colonies in solid agar plates of about 40% compared to control conditions [33].

**Figure 3:**
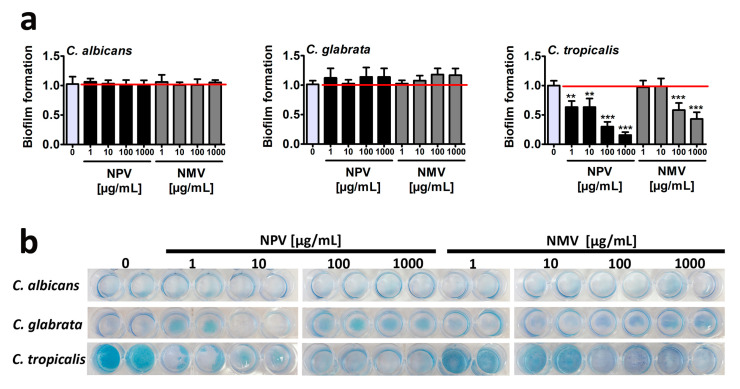
NPV- and NMV-mediated changes in biofilm formation based on *C. albicans*, *C. glabrata* and *C. tropicalis* cells. (**a**) Biofilm formation was visualized using methylene blue staining and densitometry analysis was performed using GelQuantNET software. Biofilm formation at standard growth conditions was considered as 1.0. To emphasize snake venom action, a red horizontal line is added. Bars indicate SD, *n* = 3, *** *p* < 0.001, ** *p* < 0.01 compared to the control (ANOVA and Dunnett’s a posteriori test). (**b**) Representative photographs are presented. NPV, *N. pallida* venom; NMV, *N. mossambica* venom.

Biofilms, highly structured and differentiated matrix-enclosed and surface-attached bacterial or fungal communities, display phenotypic and molecular features (e.g., gene expression patterns) that differ from their planktonic counterparts [34,35,36]. It is widely accepted that biofilm formation may promote *Candida* pathogenicity and confer resistance to both host immune cells and antimicrobial agents that can be up to 1000-fold higher than that of free-floating planktonic cells [37,38]. Thus, NPV- and NMV-induced anti-biofilm activity was then studied (Figure 3).

Surprisingly, different ability to from biofilm was revealed in three *Candida* species in control growth conditions and *C. tropicalis* was found to be the most effective in terms of biofilm formation compared to two other *Candida* species (Figure 3b). NPV and NMV did not affect the ability to form biofilm based on *C. albicans* and *C. glabrata* cells (Figure 3). In contrast, NPV and NMV had anti-biofilm activity when considering *C. tropicalis* cells (Figure 3). The anti-biofilm activity of NPV was much more pronounced than anti-biofilm activity of NMV (Figure 3a). NPV inhibited *C. tropicalis* biofilm formation when used at relatively low concentration of 1 µg/mL (about two-fold decrease in biofilm formation compared to control conditions, *p* < 0.01, Figure 3a), whereas similar effects in the case of NMV were observed when the concentration of 100 µg/mL was used (*p* < 0.001, Figure 3a). Moreover, 1 mg/mL NPV almost completely inhibited *C. tropicalis* biofilm formation (*p* < 0.001, Figure 3a). Data on snake venom-mediated anti-biofilm activity are limited to bacterial biofilms [19,39,40]. It has been reported that the protein fraction F_2_ from *N. ashei* venom exhibited anti-biofilm activity using *Staphylococcus epidermidis* cellular model [19]. The protein fraction F_2_ contained five main components, namely PLA_2_, 3FTx proteins, LAAOs, Ig-like proteins and CRISPs [19]. The authors concluded that PLA_2_, 3FTxs and LAAOs may promote anti-biofilm activity of *N. ashei* venom as they have been previously reported to possess bacteriostatic and bactericidal properties [19]. It is difficult to speculate which one component is responsible for increased anti-biofilm activity of NPV compared to NMV using *C. tropicalis* cellular model (Figure 3). NPV contains higher levels of 3FTx proteins compared to NMV (Figure 1), however antifungal activity of 3FTxs has not been reported so far. There is only one report on the effect of crotamine, one of the main constituents of the venom of the South American rattlesnake *C. durissus terrificus,* on biofilms formed by several *Candida* spp. strains, namely *C. albicans*, *C. krusei*, *C. glabrata* and *C. parapsilosis* [41]. Except minor inhibitory activity against *C. krusei* biofilm, crotamine did not affect the formation of fungal biofilms [41].

In conclusion, we considered three different methods to assess antifungal properties of crude venoms of *N. pallida* and *N. mossambica* and found limited inhibitory effects of NPV and NMV against cell viability, cell cycle and biofilm formation in three *Candida* species (Figure 2 and Figure 3). Only high concentrations of NPV and NMV (100 and/or 1000 µg/mL) affected cell viability of *C. albicans* and *C. tropicalis* (Figure 2a) and cell cycle of *C. albicans* (Figure 2b). Moreover, NPV- and NMV-induced anti-biofilm activity was observed only in the case of *C. tropicalis* (Figure 3). No inhibitory effects were noticed against *C. glabrata* cells (Figure 2 and Figure 3). Thus, one can conclude that antifungal properties of NPV and NMV against selected *Candida* species are limited (Figure 2 and Figure 3).

### 2.3. NPV- and NMV-Induced Oxidative Stress

**Figure 4:**
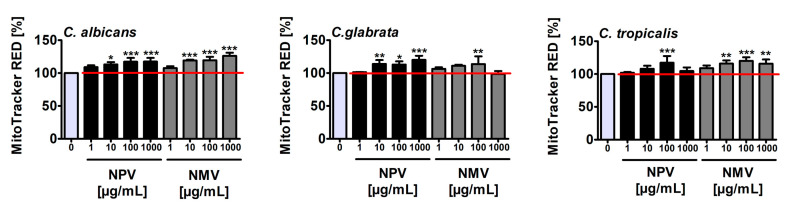
NPV- and NMV-mediated increase in the levels of mitochondrial ROS in: *C. albicans* cells (left); *C. glabrata* cells (middle); and *C. tropicalis* cells (right). Mitochondrial ROS levels were measured using ROS-specific fluorescent probe MitoTracker^®^ Red CM-H_2_Xros. Fluorescence intensity due to oxidation of reduced MitoTracker^®^ to oxidized MitoTracker^®^ was measured using a Tecan Infinite^®^ M200 fluorescence mode microplate reader. Mitochondrial ROS levels at standard growth conditions (untreated control) are considered as 100%. To emphasize snake venom action, a red horizontal line is added. Bars indicate SD, *n* = 3, *** *p* < 0.001, ** *p* < 0.01, * *p* < 0.05 compared to the control (ANOVA and Dunnett’s a posteriori test). NPV, *N. pallida* venom; NMV, *N. mossambica* venom.

As cytotoxic effects of snake venoms may be mediated by oxidative stress in different animal and human cellular models in vitro and in vivo [42,43,44,45,46,47], we then decided to analyze if NPV- and NMV-mediated changes in cell viability of *Candida* species (Figure 2a) may be accompanied by increased levels of mitochondrial reactive oxygen species (ROS) (Figure 4).

Treatment with NPV and NMV resulted in an increase of mitochondrial ROS levels of up to 26% compared to untreated *Candida* cells (Figure 4). NPV- and NMV-promoted oxidative stress was the most evident in *C. albicans* cells compared to *C. glabrata* and *C. tropicalis* cells (Figure 4). However, NPV- and NMV-mediated increase in ROS pools (Figure 4) did not correlate with changes in cell viability (Figure 2a). NPV was found to be more cytotoxic than NMV (Figure 2a), but NPV did not promote more oxidative stress than NMV (Figure 4). Moreover, treatment with NPV and NMV caused an increase in ROS levels in *C. glabrata* cells (Figure 4), but concurrently did not affect cell viability (Figure 2a). Thus, one can conclude that oxidative stress does not mediate NPV- and NMV-induced cytotoxicity in three *Candida* species (Figure 2a and Figure 4). To the best of our knowledge, there are no published data on oxidative stress-mediated snake venom cytotoxicity in *Candida* cells. It is suggested that PLA_2_ and LAAO may promote oxidative stress by their catalytic reaction, namely PLA_2_ may produce ROS during lipolysis and LAAO catalytic activity may result in the accumulation of hydrogen peroxide leading to cell death [47]. Indeed, LAAO from the viperid snake *C. rhodostoma* induced hydrogen peroxide-mediated cytotoxicity in the budding yeast *S. cerevisiae* [32]. In the present study, cobra venom-associated ROS production did not correlate with the levels of PLA_2_ and LAAO (Figure 1 and Figure 4).

### 2.4. NPV- and NMV-Mediated Secretion of Extracellular Hydrolases

**Figure 5:**
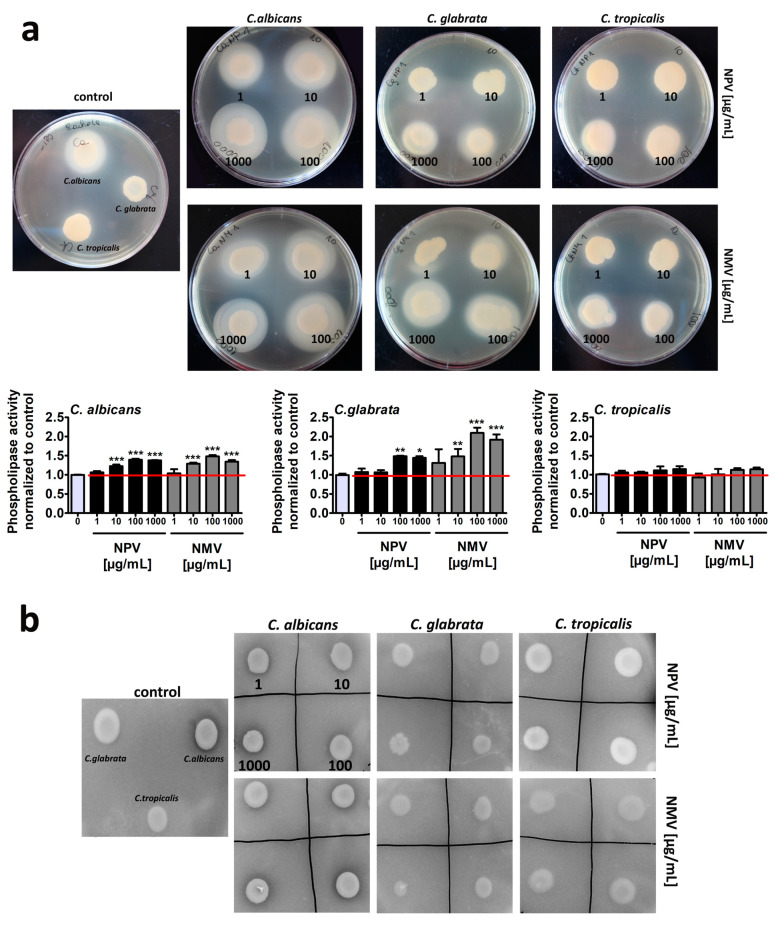
NPV- and NMV-mediated changes in the secretion of extracellular hydrolases, namely phospholipases (**a**) and secreted aspartyl proteinases (Saps) (**b**) by three *Candida* species. To reveal the secretion of extracellular hydrolases, dedicated plate-based assays were used. The phospholipase activity was judged as the formation of an opaque zone around the yeast colonies (**a**) and the proteinase activity as the formation of a transparent halo around the yeast colonies (**b**). Representative photographs are presented. Secreted phospholipase activity was measured as a diameter of an opaque zone around the yeast colonies and phospholipase activity at standard growth conditions was considered as 1.0. To emphasize snake venom action, a red horizontal line is added. Bars indicate SD, *n* = 3, *** *p* < 0.001, ** *p* < 0.01, * *p* < 0.05 compared to the control (ANOVA and Dunnett’s a posteriori test). NPV, *N. pallida* venom; NMV, *N. mossambica* venom.

The production and secretion of phospholipases and proteinases, significant virulence attributes of *Candida* pathogenicity, may facilitate fungal invasion by destroying phospholipid and protein components of host cell membranes [48,49]. Indeed, the activity of extracellular phospholipases and proteinases was reported to be correlated with *C. albicans* invasion, colonization and pathogenicity in HIV seropositive and cancer patients [50]. Extracellular phospholipases and proteinases were also considered as targets for antifungal therapy and their inhibitors were established to be potent anti-candidal and anti-cryptococcal agents in different experimental settings [51,52]. Thus, we then evaluated NPV- and NMV-mediated secretion of extracellular phospholipases and proteinases, namely secreted aspartyl proteinases (Saps) (Figure 5).

Of course, to exclude the effects of hydrolytic enzymes present in the venoms, fungal cells were treated with NPV and NMV for 2 h and venoms were then removed by three washing steps. Thus, there were no venom residues in test agar plates. In control growth conditions, *C. albicans* cells were characterized by higher phospholipase (Figure 5a) and proteinase (Figure 5b) activity compared to *C. glabrata* and *C. tropicalis* cells. This observation is in agreement with previously published data that non-*C. albicans Candida* (NCAC) species may produce extracellular phospholipases and Saps but at significantly lower levels compared to *C. albicans* [48,53,54,55]. For example, low aspartyl protease and very low phospholipase activities were detected in *C. tropicalis* isolates compared to *C. albicans* cells [55]. Surprisingly, NPV and NMV promoted the secretion of extracellular phospholipases in three *Candida* species, namely the following ranking was revealed: *C. glabrata* > *C. albicans* > *C. tropicalis* (Figure 5a). Similar effects were not observed in the case of Saps (Figure 5b). Thus, one can conclude that NPV and NMV may stimulate some pathogenic features (phospholipase activity) of the three *Candida* species used (in this study) that may limit the usefulness of the selected snake venoms, namely NPV and NMV, as antifungal agents in targeted therapy of candidiasis. Indeed, *C. glabrata* cells, characterized by the most pronounced phospholipase activity upon cobra venom treatment (Figure 5a), were also the least susceptible to NPV and NMV stimulation (Figure 2a).

### 2.5. NPV and NMV Affect Ammonia Pulses during Colony Development and Aging

**Figure 6:**
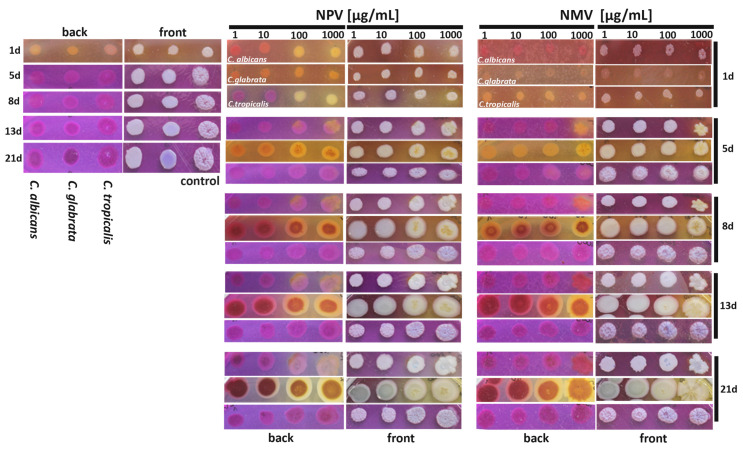
NPV- and NMV-mediated changes in ammonia pulses during colony development and aging in three *Candida* species. *Candida* cells were cultured onto bromocresol purple plates for 21 days and the phases of acidification (pH ~ 5.2, yellow color) and alkalization (pH ~ 6.8, violet color) were monitored after 1, 5, 8, 13 and 21 days of culture. Representative photographs are presented (both front and back plate view). NPV, *N. pallida* venom; NMV, *N. mossambica* venom.

Data on the effects of replicative or chronological age on *Candida* pathogenicity are limited [56]. The virulence of older populations of *C. glabrata* was potentiated compared to younger *C. glabrata* cells as judged using the *Galleria mellonella* infection model [56]. Older fungal cells were also less sensitive to oxidative stress conditions and less susceptible to neutrophil killing compared to younger counterparts [56]. Thus, it was suggested that replicative aging may be implicated in the transition from a commensal to a pathogen state in opportunistic and pathogenic fungi [56]. As fungal response to NPV and NMV may be considered complex, as both advantageous (Figure 2, Figure 3 and Figure 4) and disadvantageous (Figure 5) effects of NPV and NMV were observed in three *Candida* species in terms of antifungal therapy, we then studied other biological activities of NPV and NMV, namely their effects on quorum-sensing signal (ammonia pulses) during colony development and aging of *Candida* cells (Figure 6).

Ammonia signals are involved in colony development, metabolic reprogramming and cell differentiation of different fungal species [57,58,59,60]. For example, ammonia pulses are required for metabolic reprogramming and differentiation of chronologically aged yeast cells (elders) within a colony [58,59]. The acid-to-alkali transition between seven and ten days of colony development is associated with the occurrence of two subpopulations of elders, U cells (upper fraction cells characterized by stress resistance and longevity phenotype) and L cells (lower fraction less adapted cells being a feeding layer for U cells) [58,59]. We used a pH indicator, namely bromocresol purple plate assay, to track NPV- and NMV-mediated changes in the phases of acidification (pH ~ 5.2, yellow color) and alkalization (pH ~ 6.8, violet color) of the medium at different time points of culture (from day 1 to day 21) (Figure 6, both back and front view plates are presented). After 24 h of cell culture in control growth conditions, we were able to observe first acidic phase followed by second alkali phase after five days of culture of three *Candida* species (Figure 6). This is in agreement with previously published data on the phases of *S. cerevisiae* colony development [61]. Short initial alkali period (first alkali phase) [61] was not documented because the first photograph was taken after 24 h of culture (Figure 6). We also did not notice the second acidic phase that may be due to experiment termination after 21 days of culture or differences between colony development in *S. cerevisiae* [61] and *Candida* species (in this study). NPV and NMV affected the cycles of acidification and alkalization of the medium (Figure 6). NPV, when used at low concentrations of 1 and 10 µg/mL, accelerated second alkali phase as NPV treatment results in the occurrence of ammonia signals after 24 h of cell culture of *C. tropicalis* (Figure 6). In contrast, second alkali phase was delayed in *C. glabrata* cells treated with NPV and NMV, especially at the concentrations of 100 and 1000 µg/mL (Figure 6). The second alkali phase was also interrupted by the treatment of *C. albicans* cells with 100 and 1000 µg/mL NPV and 1000 µg/mL NMV (Figure 6). NPV and NMV also affected cell pigmentation of *C. glabrata* (Figure 6). Taken together, we documented that NPV and NMV may modulate the process of colony development and cell differentiation during colony aging and other acid-to-alkali transition-mediated processes in *Candida* species. Indeed, ammonia pulses may contribute to the pathogenesis of *C. albicans* [62]. The release of ammonia from *C. albicans* cells produced during the breakdown of amino acids promoted a yeast-to-hyphae switch, a critical virulence trait [62]. Thus, NPV- and NMV-mediated interruption or delay of second alkali phase may also limit pathogenic potential of *C. albicans* and *C. glabrata* cells (Figure 6).

## 3. Conclusions

We characterized the proteome of the two venoms of *N. pallida* and *N. mossambica* and analyzed the antifungal properties of crude venoms against three *Candida* species, namely *C. albicans*, *C. glabrata* and *C. tropicalis*. A complex response to NPV and NMV treatments was revealed. NPV and NMV affected cell viability of *C. albicans* and *C. tropicalis*, cell cycle of *C. albicans* and biofilm formation of *C. tropicalis* cells. NPV and NMV also induced oxidative stress and modulated ammonia pulses during colony development and differentiation in three *Candida* species. All these biological activities of NPV and NMV may be useful in terms of antifungal therapies. However, NPV and NMV also stimulated the secretion of extracellular phospholipases that may facilitate *Candida* pathogenicity and limit their applications as anti-candidal agents. This suggests that special care should be taken when analyzing antifungal properties of snake venoms based on limited number of pathogenic biomarkers considered.

## 4. Materials and Methods

### 4.1. Venom Collection and LC-MS/MS Analysis

Two African cobra species were considered, namely red spitting cobra *N. pallida* and Mozambique spitting cobra *N. mossambica* (Elapidae family), and imported from Tanzania and South Africa, respectively, to the Viperafarm Ltd. breeding facility (Trnava, Slovakia). Snake venoms were then collected by a specialist with SNTC/FAGASD certificates, Swaziland, Africa at the Viperafarm, with the registration number CHNZ-TT-01 granted by the state veterinary authority RVPS SR under Decision No. 05/001921 according to the Act of the National Council of the Slovak Republic No. 488/2002 Coll as described comprehensively elsewhere [15]. Briefly, venom samples were diluted 500 times in 100 mM NH_4_HCO_3_ (Merck KGaA, Darmstadt, Germany). Total protein concentration was measured using Pierce^TM^ BCA Protein Assay Kit (Thermo Fisher Scientific, Waltham, MA, USA). Protein samples (50 µg) were dried using a SpeedVac Vacuum Concentrator (Thermo Fisher Scientific, Waltham, MA, USA). Proteins were reduced using 5 mM tris-2-carboxyethyl-phosphine (TCEP, Merck KGaA, Darmstadt, Germany) at 60 °C for 1 h. Reduced cysteines were then blocked using 10 mM *S*-methyl methanethiosulfonate (MMTS, Merck KGaA, Darmstadt, Germany) at room temperature for 10 min. Trypsin (Promega, Madison, WI, USA) was then added at a 1:25 *v*/*v* ratio and samples were incubated overnight at 37 °C. Finally, to inactivate trypsin, 0.1% trifluoroacetic acid (TFA, Merck KGaA, Darmstadt, Germany) was added. Peptide identification and absolute quantitation were performed using liquid chromatography with tandem mass spectrometry (LC-MS/MS). Q Exactive mass spectrometer (Thermo Fisher Scientific, Waltham, MA, USA) coupled with a nanoACQUITY UPLC system (Waters Corporation, Milford, MA, USA) was used. Samples were applied to the nanoACQUITY UPLC Trapping Column (Waters Corporation, Milford, MA, USA) using water containing 0.1% formic acid (Merck KGaA, Darmstadt, Germany) as the mobile phase and then to the nanoACQUITY UPLC BEH C18 Column (Waters Corporation, Milford, MA, USA, 75 µm inner diameter; 250-mm long) using an acetonitrile gradient (5–35% acetonitrile for 160 min, Merck KGaA, Darmstadt, Germany) in the presence of 0.1% formic acid using a flow rate of 250 nl/min. HCD fragmentation was applied. Up to 12 MS/MS events were considered per each MS scan and the spectrometer resolution was set to 17,500. Samples were subjected to LC-MS/MS analysis in Mass Spectrometry Laboratory, Institute of Biochemistry and Biophysics, Polish Academy of Sciences, Warsaw, Poland. The acquired MS/MS raw data files were analyzed using MaxQuant software (ver. 1.6.7.0, Jürgen Cox, Max Planck Institute of Biochemistry, Martinsried, Germany) [63]. Protein identification was conducted according to UniProtKB Serpentes database (release 9/2019) using Andromeda engine. Methylthio (C) was set as fixed modification while oxidation (M) and acetyl (protein N-term) was used as variable modifications. Mass tolerance was set to 20 ppm for initial MS search, 4.5 ppm for main MS search and 20 ppm for MS/MS fragment ions. Trypsin with full specificity and maximum two missed cleavages was applied for enzyme properties. PSM and protein False Discovery Rate (FDR) were set to 1%. Hits that were identified only by site; found in decoy or contaminant lists were subsequently filtered out. At minimum, two peptides were prerequisite for protein identification and only proteins that were identified in at least three venom samples of the same species were subjected to further quantitative and qualitative proteome analysis. At this step, all hits were manually revised for proteins that were unlikely to be the components of snake venoms and filtered out. iBAQ (intensity-based absolute quantification) values of razor and unique peptides were used for the calculation of the amount of particular protein in the sample. Non-zero iBAQ values from three to six samples were averaged and attributed to specific proteins. Then, the proteins were assigned to different protein groups and the percentage of each protein group was calculated by dividing the summed iBAQ values of proteins assigned to the group by the summed iBAQ values of all quantified proteins identified in the sample. All data from LC-MS/MS analyses were deposited in the PRIDE repository: Project accession: PXD020450; Project DOI: 10.6019/PXD020450. Submitted data include raw data from the instrument as well as result mzTab files from MaxQuant with all spectra attached for *N. mossambica* and *N. pallida* samples. Submitted data also contain a list of all identified peptides, file with the list of identified protein groups and a separate file for parameters of MaxQuant analyses. All the calculated data with particular pie charts were placed into the final Excel file which can be accessed from PRIDE but also directly from the Supplementary Materials. Detailed data processing protocol is available in this subsection (above) as well as in the description in the PRIDE repository.

### 4.2. Candida Species and Culture Conditions

Two clinical *Candida* species isolates, namely *Candida glabrata* 4246 and *Candida tropicalis* 4114, were obtained from the Clinical Microbiology Laboratory (Department of Diagnostic Medicine, Provincial Medical Specialist Unit, Rzeszow, Poland) and comprehensively characterized elsewhere [64]. This study was approved by the Ethics Committee of the Faculty of Medicine, University of Rzeszow, Poland (approval code 2018/06/03, approved on 14 June 2018). The reference strain of *Candida albicans* (Robin) Berkhout (ATCC^®^ 14053^™^) was obtained from American Type Culture Collection (ATCC, Manassas, VA, USA). *Candida* cells originated from a single colony were routinely propagated using liquid yeast extract peptone dextrose (YPD) medium (1% *w*/*v* Difco Yeast Extract, 2% *w*/*v* Difco Yeast Bacto-Peptone, 2% *w*/*v* dextrose) (BD Biosciences, Sparks, MD, USA) with shaking at 30 °C. *Candida* cells at logarithmic phase of growth were treated with cobra venoms (*N. pallida* venom, NPV and *N. mossambica* venom, NMV) at the concentrations of 1, 10, 100 and 1000 µg/mL for 2 h, and then cobra venoms were removed and *Candida* cells were cultured without cobra venoms for 24/48/72 h (majority of assays) and up to 21 days (colony development and aging assay). The rationale to consider four diverse concentrations of snake venoms was the evaluation of the antifungal activity of snake venoms at very low (1 µg/mL), low (10 µg/mL), relatively high (100 µg/mL) and high (1000 µg/mL) concentrations. We are aware that the treatment with 1000 µg/mL snake venoms might have limited clinical relevance. Moreover, short-term treatment was used to elucidate if snake venoms might promote cytotoxic effects when *Candida* cells were stimulated for only 2 h.

### 4.3. Resazurin-Based Analysis of Metabolically Active Cells and Cell Viability

*Candida* cells at logarithmic phase of growth (1 × 10^6^ cells/mL) were treated with NPV or NMV (1, 10, 100 and 1000 µg/mL) for 2 h; the cells were then washed, resuspended in YPD medium and cultured in a 96-well format incubator with shaking at 30 °C for 24 h. Ten microliters of 0.2 mg/mL resazurin (7-hydroxy-3H-phenoxazin-3-one 10-oxide) (Merck KGaA, Darmstadt, Germany) dissolved in phosphate buffer saline (PBS) were added to each well. The plates were then incubated at 30 °C for 1 h. Resazurin is reduced to pink-colored resorufin (7-hydroxy-3H-phenoxazin-3-one) by mitochondrial oxidoreductases of metabolically active cells that may be considered as an indicator of mitochondrial metabolic activity and cell viability. The resulting absorbance was measured using a Tecan Infinite^®^ M200 (Tecan Group Ltd., Männedorf, Switzerland) absorbance mode microplate reader at 570 nm.

### 4.4. Cell Cycle

*Candida* cells at logarithmic phase of growth (1 × 10^6^ cells/mL) were treated with NPV or NMV (1, 10, 100 and 1000 µg/mL) for 2 h; the cells were then washed, resuspended in TE buffer (50 mM Tris-HCl, pH 8 containing 50 mM EDTA) and fixed using 95% ethanol. Cells were then washed using TE buffer and treated with 1 mg/mL RNAse A at 37 °C for 1 h and then treated with 5 mg/mL Proteinase K at 37 °C for 1 h. Cells were then washed using TE buffer and resuspended in a SYBR Green I solution (1:85 dilution of a commercial stock in TE buffer, Thermo Fisher Scientific, Waltham, MA, USA) and incubated overnight at 4 °C. Stained cells were then resuspended in TE buffer, and the phases of cell cycle (G1, S and G2/M) were analyzed using an Amnis^®^ FlowSight^®^ (Merck KGaA, Darmstadt, Germany) imaging flow cytometer and IDEAS software version 6.2.187.0 (Merck KGaA, Darmstadt, Germany). Representative histograms (normalized frequency vs intensity) are presented.

### 4.5. Biofilm Formation

*Candida* cells at logarithmic phase of growth (1 × 10^6^ cells/mL) were treated with NPV or NMV (1, 10, 100 and 1000 µg/mL) for 2 h, washed and resuspended in YPD medium (a total volume of 500 μL per well of a 24-well plate) and cultured in a 24-well format incubator with shaking at 37 °C for 48 h. YPD medium was then removed; cells were then washed twice using PBS to remove loosely adherent cells and biofilm formation was revealed using methylene blue staining (1:10,000 dilution of 0.01% stock solution in PBS). Plates were photographed and densitometry analysis was conducted using GelQuantNET software (version 1.8.2, Biochemlabsolutions.com, University of California, San Francisco, CA, USA). Biofilm formation at standard growth conditions was considered as 1.0. Data (biofilm formation) were normalized to control.

### 4.6. Mitochondrial ROS Levels

Mitochondrial ROS levels were measured using ROS-specific fluorescent probe, namely MitoTracker^®^ Red CM-H_2_Xros (Thermo Fisher Scientific, Waltham, MA, USA). *Candida* cells at logarithmic phase of growth (1 × 10^6^ cells/mL) were treated with NPV or NMV (1, 10, 100 and 1000 µg/mL) for 2 h; washed; suspended in PBS containing 0.1% glucose, 0.5 mM EDTA and 30 nM MitoTracker^®^ Red CM-H_2_Xros; and incubated in the dark at 30 °C for 30 min. Fluorescence intensity due to oxidation of reduced MitoTracker^®^ to oxidized MitoTracker^®^ was measured using a Tecan Infinite^®^ M200 fluorescence mode microplate reader. Measurement conditions were: λ_ex_ = 579 nm and λ_em_ = 599 nm. Mitochondrial ROS levels at standard growth conditions (untreated control) are considered as 100%.

### 4.7. Secretion of Extracellular Hydrolases

Secretion of exoenzymes, namely phospholipases and secreted aspartyl proteinases (Saps) was evaluated using agar plate-based methods [65,66] with minor modifications [67]. Namely, for phospholipase assay, Sabouraud dextrose agar (SDA) containing 1 M NaCl, 5 mM CaCl_2_ and 4% (*v*/*v*) sterile egg yolk (BD Biosciences, Sparks, MD, USA) was used and for proteinase assay; 60 mL of solution containing MgSO_4_ × 7H_2_O (0.04 g), K_2_HPO_4_ (0.5 g), NaCl (1 g), yeast extract (0.2 g), glucose (4 g) and bovine serum albumin (BSA, 0.5 g) (Merck KGaA, Darmstadt, Germany), pH 3.5, was added to 140 mL of 3% (*v*/*v*) agar. *Candida* cells at logarithmic phase of growth (1 × 10^6^ cells/mL) were treated with NPV or NMV (1, 10, 100 and 1000 µg/mL) for 2 h, washed and resuspended in YPD medium. Ten microliters of *Candida* cell suspensions (1 × 10^6^ cells/mL) were inoculated onto phospholipase and proteinase assay solid agar plates and incubated at 37 °C for 72 h. The phospholipase activity (secreted exoenzyme activity) in the culture plate was judged as the formation of an opaque zone around the yeast colonies and the proteinase activity (secreted exoenzyme activity) as the formation of a transparent halo around the yeast colonies [66]. Plates were photographed and secreted phospholipase activity was measured as a diameter of an opaque zone around the yeast colonies and phospholipase activity at standard growth conditions was considered as 1.0. Data (phospholipase activity) were normalized to control.

### 4.8. Ammonia Pulses during Colony Development and Aging

*Candida* cells at logarithmic phase of growth (1 × 10^6^ cells/mL) were treated with NPV or NMV (1, 10, 100 and 1000 µg/mL) for 2 h, washed and resuspended in YPD medium. Then, 2 μL of cell suspensions were inoculated onto colony development and aging assay medium (1% yeast extract, 3% glycerol, 30 mM CaCl_2_, 0.01% bromocresol purple, 2% agar) and incubated at 30 °C for up to 21 days. Plates were photographed and acid-to-alkali transition was monitored after 1, 5, 8, 13 and 21 days of culture.

### 4.9. Statistical Analysis

The mean values ± SD were calculated on the basis of at least three independent experiments. Statistical significance was evaluated using GraphPad Prism 5 (GraphPad Software, San Diego, CA, USA) using one-way ANOVA and Dunnett’s test.

## Supplementary Materials

The following are available online at https://www.mdpi.com/2072-6651/12/8/500/s1, Supplementary Excel File: A list of identified proteins and peptides of *N. pallida* and *N. mossambica* venoms.
